# 

**Published:** 1852-01

**Authors:** 


### CONTENTS OF THE DENTAL REGISTER OF THE WEST.

JANUARY, 1852.

PART I.—ORIGINAL COMMUNICATIONS.

PAGE.
Dr. James Taylor's Introductory Address, .................... 65
Atmospheric Pressure Plates, by Dr. J. P Ullrey, ............. 84
Dental Irritation, by H. E. Peebles, ......................... 87
Osseous Union of Teeth, by H. Scott, ......................... 91
Letter of A. Berry, ........................................... 92
Letter on the Insertion and Preparation of Natural Teeth, ...... 94

PART II.—SELECTIONS.

A new mode of Setting Artificial Teeth, ..................... 95
Artistic Dentistry, ............................................ 97
Proceedings twelfth annual Meeting Am. S. Dental Surgeons, ... 111
A new Anæsthetic, ............................................ 117

PART III.—EDITORIAL.

Gardette's proposition to establish a Lectureship on Dental Surgery in Medical Colleges, ..................................... 119
Dental Chair, .................................................. 124
Dental Recorder, ............................................... 124
Proceedings of the Miss. Valley Association Dental Surgeons, ... 125
Ohio College Dental Surgery, ................................... 125
Stockholders Ohio College Dental Surgery, ...................... 125
College Commencement, .......................................... 125
The College Library and Museum, ................................ 126
Persons claiming to be Graduates of Ohio College Den. Sur., ... 126
To Subscribers of Dental Register, ............................. 126
Our Exchanges, ................................................. 126
Dental Depot at Louisville, .................................... 126

